# Interactive Effects of Tillage and Soil Depth on Soil Aggregate Stability in Relation to Soil Properties and Glomalin-Related Soil Proteins

**DOI:** 10.3390/biology15161404

**Published:** 2026-08-16

**Authors:** Chunjuan Wang, Xinyi Bi, Yue Yang, Yongfei Wei, Meiyu Chu, Wei Chen, Jinlong Wang, Jinwei Zhang

**Affiliations:** 1 Teaching Affairs Office of Beihua University, Institute of Higher Education, Beihua University, Jilin 132013, China; wangcj1022@beihua.edu.cn; 2 College of Science, Traditional Chinese Medicine Biotechnology Innovation Center in Jilin Province, Beihua University, Jilin 132013, China; bxyyy0605@163.com (X.B.); 15143737916@163.com (Y.Y.); wyongfei2026@163.com (Y.W.); chumeiyu2026@163.com (M.C.); chenw352@126.com (W.C.); 3 Department of Grassland Science, College of Animal Science and Technology, Northeast Agricultural University, Harbin 150030, China

**Keywords:** glomalin-related soil protein, soil structural stability, conservation tillage, Mollisol, structural equation modeling, soil profile

## Abstract

Tillage practices influence soil structural stability by modifying the soil physicochemical environment and biological processes. However, the relationships among tillage, soil depth, Glomalin-related soil proteins (GRSP), and soil aggregate stability remain insufficiently understood, particularly in the Mollisols of Northeast China. A long-term field experiment was conducted to investigate the effects of three tillage practices, including no tillage (NT), deep tillage (DT), and rotary tillage (RT), across four soil depths (0–10, 10–20, 20–30, and 30–40 cm). Soil physicochemical properties, glomalin-related soil proteins (total glomalin, TTG; easily extractable glomalin, EEG), and soil aggregate stability indices, including mean weight diameter (MWD), geometric mean diameter (GMD), and the percentage of water-stable aggregates (R_0.25_), were determined. Pearson correlation analysis and structural equation modeling (SEM) were employed to elucidate the relationships among soil physicochemical properties, GRSP, and aggregate stability. Tillage practices significantly altered soil physicochemical properties, particularly soil moisture and electrical conductivity, with responses varying across soil depths. Deep tillage generally enhanced EEG accumulation and improved aggregate stability compared with rotary tillage, whereas TTG exhibited relatively smaller variations among treatments. Soil moisture was positively correlated with GRSP and aggregate stability, whereas electrical conductivity showed predominantly negative relationships with biological indicators. Correlation analysis showed significant associations among soil physicochemical properties, GRSP, and aggregate stability. In the SEM, soil physicochemical properties were positively associated with both GRSP (standardized path coefficient = 0.205, *p* < 0.05) and aggregate stability (standardized path coefficient = 0.416, *p* < 0.05), whereas the direct pathway from GRSP to aggregate stability was not statistically significant. Tillage and soil depth were significantly associated with variation in soil physicochemical properties and GRSP, while soil depth also showed a significant direct association with aggregate stability. These results indicate that the final SEM supported a stronger statistical association of soil physicochemical properties with aggregate stability than of GRSP with aggregate stability. Although GRSP was correlated with aggregate stability in the correlation analysis, its independent contribution to aggregate stability was not supported by the final SEM. These results provide new insights into the processes associated with soil structural stabilization and offer a scientific basis for optimizing tillage management to improve soil quality and promote sustainable agricultural development in the black soil region of Northeast China.

## Simple Summary

Soil aggregate stability is an important indicator of soil quality because it affects water retention, nutrient cycling, and crop productivity. Agricultural practices, especially tillage management, can modify soil environments and influence soil structure. In this study, we investigated how three tillage practices, including no tillage, deep tillage, and rotary tillage, affected soil physicochemical properties, glomalin-related soil proteins, and aggregate stability across different soil depths in a black soil region of Northeast China. We found that tillage effects varied with soil depth, and deep tillage was associated with improved soil environmental conditions and higher aggregate stability compared with rotary tillage. Our results suggest that changes in soil conditions and biological indicators such as glomalin-related soil proteins are closely associated with soil structural variation. However, further studies involving direct measurements of microbial communities, arbuscular mycorrhizal fungi, and root traits are needed to better understand the biological mechanisms underlying soil aggregation. These findings provide useful information for improving soil management strategies in black soil agroecosystems.

## 1. Introduction

Soil aggregates are the basic structural units of soil and are essential for maintaining soil physical quality, nutrient cycling, carbon sequestration, water retention, and biological activity. Stable aggregates improve pore continuity, enhance water infiltration and aeration, reduce soil erosion, and create suitable habitats for soil microorganisms, thereby contributing to sustainable crop production and ecosystem functioning [1,2]. Aggregate stability is therefore widely recognized as one of the most important indicators of soil quality and soil health. In agricultural ecosystems, the formation and stabilization of aggregates are jointly controlled by physical, chemical, and biological processes, which are highly sensitive to land management practices [3,4]. Consequently, understanding the processes associated with soil aggregate stability has become an important topic in soil science, particularly in regions where long-term cultivation has resulted in soil structural degradation.

The black soil (Mollisol) region of Northeast China is one of the most productive agricultural areas in the world and plays a critical role in national food security. However, decades of intensive cultivation, frequent tillage, and excessive mechanical disturbance have accelerated the breakdown of macroaggregates, reduced soil organic carbon storage, and deteriorated soil physical structure [4,5]. Soil compaction, reduced infiltration capacity, and declining aggregate stability have become widespread problems in this region, threatening the sustainability of agricultural production. Various conservation tillage practices, including no tillage and deep tillage, have therefore been widely adopted to improve soil structure and restore soil functions. Nevertheless, the responses of soil aggregates to different tillage practices remain inconsistent because tillage simultaneously alters soil moisture, aeration, nutrient availability, and belowground biological processes, and these effects may vary considerably with soil depth [6,7]. Tillage management represents one of the most important anthropogenic factors influencing soil structural development in agricultural ecosystems [8]. Different tillage systems modify soil disturbance intensity, residue distribution, pore characteristics, and resource availability, thereby producing contrasting effects on soil physical quality and biological processes [7]. No tillage reduces mechanical disturbance and promotes soil structure preservation, whereas deep tillage can alleviate subsurface compaction and improve the redistribution of water and nutrients within the soil profile [9]. Rotary tillage, although commonly used for seedbed preparation, may cause repeated disturbance of soil aggregates when applied intensively [10]. However, the effects of these tillage practices on soil structural stability remain highly dependent on soil conditions and depth, and the underlying ecological pathways require further investigation.

Previous studies have demonstrated that soil aggregate formation depends not only on physical protection but also on biological binding agents produced by plant roots and soil microorganisms [11,12]. Among these biological agents, Glomalin-related soil proteins (GRSP), a group of soil proteins largely associated with arbuscular mycorrhizal fungi (AMF), has received increasing attention because of its important role in improving soil structural stability. GRSP is a relatively stable glycoprotein that can bind mineral particles, clay, and organic matter into larger aggregates while protecting organic carbon from microbial decomposition [13,14]. GRSP is commonly quantified as easily extractable glomalin (EEG) and total glomalin (TTG). EEG represents a relatively labile GRSP fraction that may respond more rapidly to environmental changes, whereas TTG represents a relatively stable GRSP pool reflecting longer-term glomalin accumulation in soils [15,16]. Numerous studies have reported significant positive relationships between GRSP concentration and aggregate stability indices, including mean weight diameter (MWD), geometric mean diameter (GMD), and the proportion of water-stable aggregates [17,18]. Therefore, GRSP has been proposed as a useful indicator associated with soil structural quality and ecosystem restoration. Tillage practices may influence GRSP accumulation by modifying soil environmental conditions associated with biological processes. Conventional rotary tillage may reduce the stability of soil aggregates through repeated disturbance, whereas no tillage and deep tillage may maintain or improve soil structural conditions through different pathways [19,20,21,22]. However, whether deep tillage promotes GRSP accumulation and aggregate stabilization across different soil depths remains unclear.

In addition to tillage-induced changes in soil structure, Soil physicochemical properties are considered important factors associated with GRSP accumulation and aggregate stability. Soil moisture affects microbial metabolism and root activity, electrical conductivity reflects soil salinity stress, and nutrient availability influences plant growth and belowground biological interactions [23,24]. These environmental factors may influence aggregate formation and may also affect GRSP accumulation, thereby indirectly affecting soil structural stability. Consequently, soil aggregate stability should be regarded as the result of multiple interacting processes rather than the consequence of a single environmental factor [25]. Although many studies have separately investigated the effects of tillage on soil physicochemical properties, GRSP, or aggregate stability, relatively few have attempted to integrate these components into a unified analytical framework capable of distinguishing direct and indirect effects.

Integrative approaches are increasingly needed to understand how agricultural management practices influence soil structural functions because aggregate stability results from the interaction of multiple physical, chemical, and biological processes [26,27]. Structural equation modeling (SEM) provides a useful statistical framework for evaluating hypothesized relationships among multiple variables and distinguishing direct and indirect associations within complex ecological systems [28]. However, the key challenge remains to understand how management-induced environmental changes are associated with biological indicators and soil structural responses, particularly across different soil depths. Such information is essential for understanding the mechanisms underlying soil structure formation and for developing effective management strategies for black soil conservation.

Previous studies have mainly focused on the individual effects of tillage practices, soil physicochemical properties, GRSP, or aggregate stability, while the depth-dependent relationships among these components remain insufficiently understood, particularly in Mollisol agroecosystems [29,30]. Therefore, the objectives of the present study were to investigate the effects of three tillage practices (no tillage, deep tillage, and rotary tillage) on soil physicochemical properties, glomalin-related soil proteins, and aggregate stability across four soil depths (0–10, 10–20, 20–30, and 30–40 cm) in a Mollisol of Northeast China. Pearson correlation analysis was used to identify the relationships among soil physicochemical properties, GRSP, and aggregate stability, whereas structural equation modeling was employed to evaluate the direct and indirect relationships linking tillage practices, soil depth, soil properties, GRSP, and soil aggregate stability. We hypothesized that (i) tillage practices would significantly modify soil physicochemical properties in a depth-dependent manner; (ii) improvements in soil physicochemical conditions would be associated with increased GRSP accumulation and enhanced soil aggregate stability; and (iii) the effects of tillage on soil aggregate stability would be closely associated with changes in soil physicochemical properties and GRSP rather than through direct effects alone. The findings of this study provide new insights into the biological processes associated with soil aggregate stabilization under different tillage systems and offer a theoretical basis for optimizing tillage management in the black soil region of Northeast China.

## 2. Materials and Methods

### 2.1. Study Site

The field experiment was conducted at the Experimental Station of Beihua University located in Nong’an County, Jilin Province, Northeast China (approximately 44°26′ N, 125°10′ E). The study site is located in the central part of the Songnen Plain, which represents one of the major Mollisol regions in Northeast China. The region has a temperate continental monsoon climate, characterized by cold and dry winters and warm and humid summers. The mean annual temperature is approximately 5.0–5.5 °C, with monthly mean temperatures ranging from approximately −18 °C in January to 23 °C in July. The average annual precipitation is 500–550 mm, with approximately 70–80% occurring between June and September during the maize growing season. The annual frost-free period is approximately 145–155 days, and the annual sunshine duration exceeds 2600 h. According to the World Reference Base for Soil Resources (IUSS Working Group WRB), the experimental soil was classified as a Phaeozem, corresponding to the typical black soils (Mollisols) distributed in Northeast China. The soil represents a highly productive agricultural soil characterized by relatively high organic matter content and favorable soil fertility conditions. Before the establishment of the tillage treatments, the field had been continuously cultivated with maize under conventional agricultural management practices. Prior to the experiment, soil samples were collected from the 0–40 cm soil layer to characterize the baseline soil properties. The mean soil organic matter content was 104.37 g kg^−1^, and the mean soil pH was 7.30. The concentrations of ammonium nitrogen (NH_4_^+^-N), available phosphorus (AP), and available potassium (AK) were 35.34, 56.78, and 211.23 mg kg^−1^, respectively. The mean soil moisture and electrical conductivity were 16.34% and 122.75 μS cm^−1^, respectively. These baseline characteristics confirmed that the experimental site represented a typical fertile Mollisol agroecosystem in Northeast China. The maize cultivar used in this experiment was Zhengdan 958, a widely cultivated hybrid maize variety in Northeast China. The cultivar was selected because of its broad adaptation to local climatic conditions and its extensive application in regional maize production systems.

### 2.2. Experimental Design and Soil Sampling

A randomized complete block design (RCBD) was established in 2020 at the experimental station of Beihua University. The experiment consisted of three tillage treatments: no tillage (NT), deep tillage (DT), and rotary tillage (RT). Each treatment included five independent replicate plots, resulting in a total of 15 experimental plots. The tillage treatments were maintained under the same fertilization regime and crop management practices throughout the experimental period. In the NT treatment, maize was cultivated using the original ridge system without mechanical soil disturbance. The DT treatment involved deep soil loosening toa depth of approximately 40 cm, whereas the RT treatment involved rotary tillage to a depth of approximately 15 cm before maize sowing. Maize (*Zea mays* L., cultivar Zhengdan 958) was cultivated annually as a continuous maize monoculture system using local agronomic practices. The experiment was maintained under identical fertilization and crop management practices throughout the experimental period to ensure that differences among treatments were mainly associated with tillage management.

Maize (*Zea mays* L., cultivar Zhengdan 958) was grown annually as a continuous maize monoculture system using local agronomic practices. The field had been continuously cultivated with maize before and during the experimental period. The maize crop was harvested in early October 2023, and soil samples were collected immediately after harvest to minimize temporal variation associated with crop growth.

For each replicate plot, four soil cores were randomly collected from the 0–40 cm soil profile. Samples were separated into four soil layers (0–10, 10–20, 20–30, and 30–40 cm), and soil samples from the same depth within each plot were thoroughly mixed to obtain one composite sample. A total of 60 soil samples were collected (3 tillage treatments × 4 soil depths × 5 replicates).

Fresh soil samples were transported to the laboratory in insulated containers. Visible plant residues, roots, and stones were manually removed, and the samples were gently passed through a 2-mm sieve. Each sample was divided into three subsamples: one was stored at 4 °C for glomalin-related soil protein determination, one was air-dried for soil physicochemical property and aggregate stability analyses, and the remaining portion was used for soil moisture determination.

### 2.3. Determination of Soil Physicochemical Properties

Soil moisture (SM) was determined gravimetrically by drying fresh soil at 105 °C to constant weight [31]. Soil pH was measured in a soil-to-water suspension (1:2.5, *w*/*v*) using a digital pH meter. Electrical conductivity (EC) was determined using a conductivity meter after extraction with deionized water. Soil organic matter (SOM) was determined using the potassium dichromate oxidation method [32]. Available phosphorus (AP) was extracted with 0.5 mol L^−1^ NaHCO_3_ solution and quantified using the molybdenum blue colorimetric method [33]. Available potassium (AK) was extracted with 1 mol L^−1^ ammonium acetate and measured using a flame photometer [33]. Ammonium nitrogen (NH_4_^+^–N) was determined by continuous-flow analysis following KCl extraction [33]. Soil salt content (SSC) was determined using the residue-drying method after water extraction [34]. All analyses were performed according to the standard procedures recommended by the Chinese Society of Soil Science.

### 2.4. Determination of Glomalin-Related Soil Protein (GRSP)

GRSP was determined following the citrate extraction method originally described by Wright and Upadhyaya [35]. EEG was extracted from 1.0 g of soil using 20 mmol L^−1^ sodium citrate buffer (pH 7.0) by autoclaving at 121 °C for 30 min. TTG was extracted using 50 mmol L^−1^ sodium citrate buffer (pH 8.0) through repeated autoclaving at 121 °C for 60 min until the supernatant became colorless. After centrifugation, the supernatants were collected and protein concentrations were determined using the Bradford assay with bovine serum albumin as the standard. GRSP concentrations were expressed as mg g^−1^ dry soil.

### 2.5. Soil Aggregate Stability Analysis

Water-stable aggregates were determined using the wet-sieving method [36]. Air-dried soil samples (100 g) were placed on a nest of sieves with mesh sizes of 2.0, 0.9, 0.5, and 0.1 mm and oscillated vertically in water for 30 min. The soil retained on each sieve and the fraction passing through the 0.1-mm sieve were collected, oven-dried, and weighed. The water-stable aggregates were classified into five size fractions: >2 mm, 0.9–2 mm, 0.5–0.9 mm, 0.1–0.5 mm, and <0.1 mm. Aggregate stability was further evaluated using: Total glomalin (TTG), Easily extractable glomalin (EEG), Mean weight diameter (MWD), Geometric mean diameter (GMD), Aggregate stability ratio (R). MWD and GMD were calculated according to the following equations: $MWD=\sum x_{i}w_{i}$, $GMD=\exp(\sum W_{i}lnX_{i})$, where (*X_i_*) is the mean diameter of each aggregate fraction and (*W_i_*) is its corresponding mass proportion.

### 2.6. Statistical Analysis

All statistical analyses were performed using R software (version 4.3.2). Because four soil depths were sampled within each experimental plot, linear mixed-effects models were used to evaluate the effects of tillage, soil depth, and their interaction on soil physicochemical properties, glomalin-related soil protein (GRSP) fractions, aggregate-size distribution, and aggregate stability indices. Tillage, soil depth, and their interaction were treated as fixed effects, with experimental plot included as a random effect to account for the dependence among soil depths. Significant differences were evaluated using Tukey-adjusted pairwise comparisons at *p* < 0.05.

Pearson correlation analysis was used to examine relationships among soil physicochemical properties, GRSP, and aggregate stability indices, and correlations were visualized using the corrplot package (version 0.92). Principal component analysis (PCA) was used to characterize the major variation in soil physicochemical properties and assist variable selection for structural equation modeling (SEM). Eight variables (SM, EC, SOM, NH_4_^+^-N, AP, AK, pH, and SSC) were initially considered. Based on their PCA contributions and associations with GRSP and aggregate stability in the correlation analysis, SM and EC were retained as representative soil physicochemical variables, thereby reducing redundancy and potential multicollinearity. The PC1 scores derived from TTG and EEG and from MWD, GMD, and R were used to represent GRSP and aggregate stability, respectively.

SEM was performed using the piecewiseSEM package (version 2.3.1) to evaluate the hypothesized relationships among tillage, soil depth, soil physicochemical properties, GRSP, and aggregate stability. Experimental plot was included as a random effect in the component models where appropriate. Model fit was evaluated using Fisher’s C statistic and the Chi-square test, and standardized path coefficients were used to describe the strength and direction of the relationships. The analytical framework was adapted from our previous study [37].

Permutational multivariate analysis of variance (PERMANOVA) was used to assess the effects of tillage practices, soil depth, and their combined effects on the overall variation in soil physicochemical properties. Prior to analysis, the soil physicochemical variables were standardized to account for differences in measurement units and scales. PERMANOVA was conducted on the resulting multivariate soil-property matrix, with tillage practice and soil depth included as explanatory factors. The proportion of variation explained by the model was expressed as R^2^, and statistical significance was assessed using permutation tests.

## 3. Results

### 3.1. Effects of Tillage Practices and Soil Depth on Soil Physicochemical Properties

The effects of tillage practices and soil depth on soil physicochemical properties are presented in Table 1. Linear mixed-effects models showed that tillage significantly affected SM, EC, NH_4_^+^-N, and pH (*p* < 0.05), whereas soil depth significantly affected SM, EC, NH_4_^+^-N, AP, AK, and pH (*p* < 0.05; Table S1). Significant tillage × soil depth interactions were detected for SM (F = 4.628, *p* = 0.001), EC (F = 7.815, *p* < 0.001), and AK (F = 5.025, *p* < 0.001), whereas no significant interactions were observed for SOM, NH_4_^+^-N, AP, pH, or SSC (*p* > 0.05; Table S1).

Across individual soil depths, SM was generally higher under no tillage and deep tillage than under rotary tillage, particularly at 20–30 cm. EC also varied among tillage treatments and soil depths, with deep tillage showing higher values than rotary tillage in the upper soil layers. SOM remained relatively stable across treatments and depths. AP varied primarily with soil depth, whereas the responses of AK differed among tillage treatments depending on soil depth.

**Figure 1:**
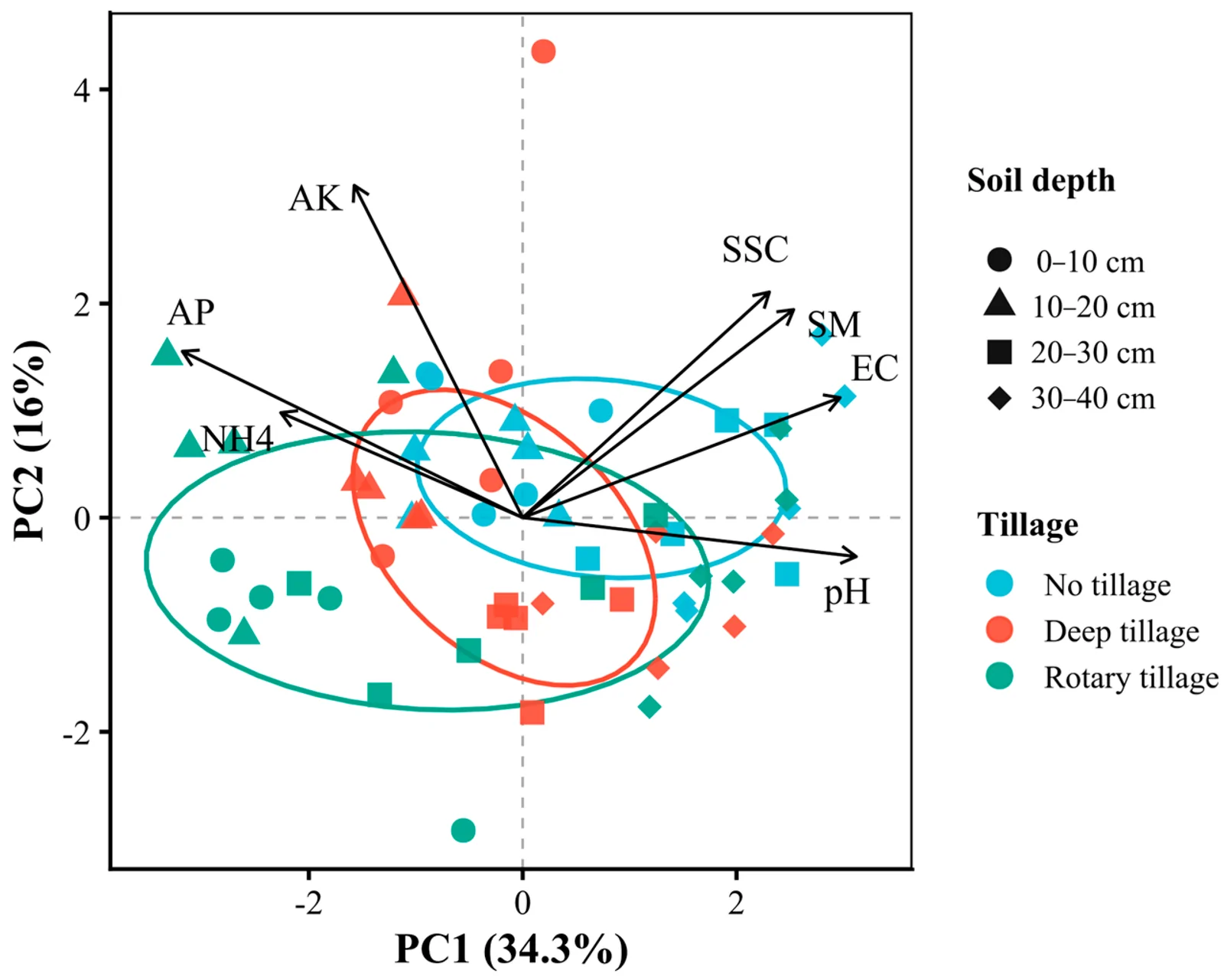
Principal component analysis (PCA) of soil physicochemical properties under different tillage practices and soil depths. Colors indicate tillage practices, symbols represent soil depths, and arrows denote soil physicochemical variables. Ellipses indicate the 60% confidence intervals for each tillage treatment.

To further characterize the overall variation in soil physicochemical properties, PCA was performed (Figure 1). The first two principal components explained 50.3% of the total variance, with PC1 and PC2 accounting for 34.3% and 16.0%, respectively. SM, EC, SSC, and pH were positively associated with PC1, whereas AK, AP, and NH_4_^+^ showed negative loadings along the same axis, suggesting that these variables represented the major environmental gradients across treatments and soil depths. Samples from different tillage practices exhibited partial separation along the first principal component, although substantial overlap among confidence ellipses indicated that tillage alone did not completely distinguish the overall soil physicochemical characteristics. In contrast, soil depth contributed substantially to sample distribution, reflecting the strong vertical differentiation of soil environmental conditions.

Permutational multivariate analysis of variance (PERMANOVA) further demonstrated that tillage practices alone did not significantly alter the overall soil physicochemical properties (R^2^ = 0.035, *p* = 0.405). However, the combined effects of tillage practices and soil depth significantly explained the variation in soil physicochemical characteristics (R^2^ = 0.517, *p* = 0.001), indicating that the responses of soil properties to tillage were strongly dependent on soil depth.

### 3.2. Responses of Glomalin-Related Soil Proteins and Soil Aggregate Stability

**Figure 2:**
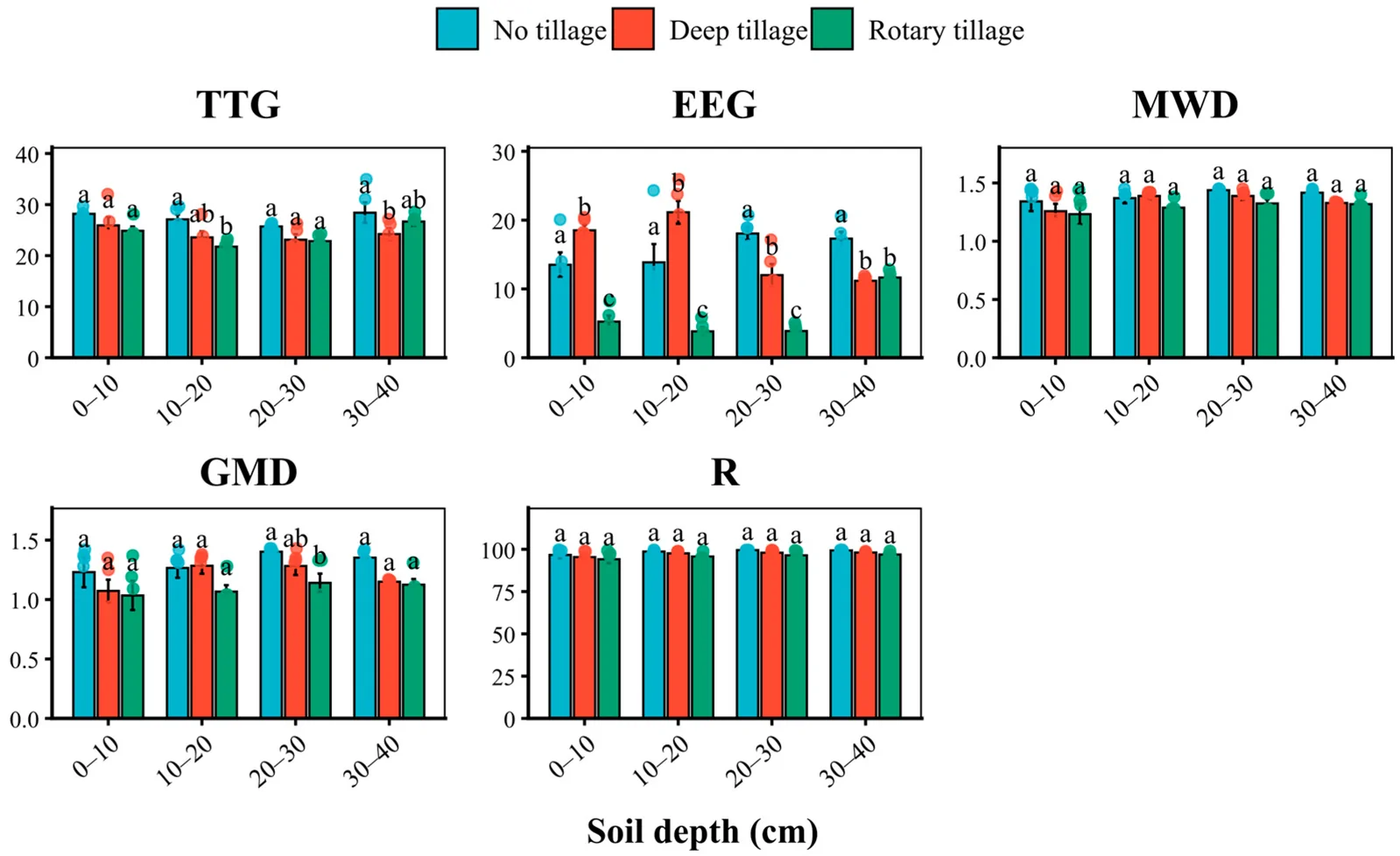
Responses of glomalin-related soil proteins and soil aggregate stability to different tillage practices across soil depths. Note: values are presented as means ± SD (n = 5). Different lowercase letters indicate significant differences among tillage treatments within the same soil depth (*p* < 0.05). TTG, total glomalin-related soil protein; EEG, easily extractable glomalin-related soil protein; MWD, mean weight diameter; GMD, geometric mean diameter; R, percentage of water-stable aggregates.

Glomalin-related soil proteins responded differently to tillage practices and soil depth (Figure 2; Table S1). Linear mixed-effects models showed significant effects of both tillage and soil depth on TTG (tillage: F = 6.750, *p* = 0.011; depth: F = 6.743, *p* = 0.001), whereas their interaction was not significant (*p* = 0.280). In contrast, EEG was significantly affected by tillage (F = 49.735, *p* < 0.001), with a significant tillage × soil depth interaction (F = 14.567, *p* < 0.001), while the main effect of soil depth was not significant (*p* = 0.182; Table S1). Across individual soil depths, deep tillage showed higher EEG contents than rotary tillage in the 0–10, 10–20, and 20–30 cm layers (*p* < 0.05), whereas at 30–40 cm, no significant difference was observed between deep and rotary tillage.

Soil aggregate stability showed variable responses to tillage and soil depth (Figure 2; Table S1). Soil depth significantly affected MWD (F = 3.304, *p* = 0.031), whereas the tillage effect did not reach statistical significance (F = 3.685, *p* = 0.057). GMD was significantly affected by tillage (F = 6.472, *p* = 0.012), whereas the effect of soil depth was not significant (*p* = 0.069). Both tillage (F = 4.183, *p* = 0.042) and soil depth (F = 3.631, *p* = 0.022) significantly affected R. No significant tillage × soil depth interactions were detected for MWD, GMD, or R (*p* > 0.05; Table S1). Pairwise comparisons within individual soil depths revealed relatively limited differences in MWD, GMD, and R among tillage treatments, and R remained consistently high (>90%) across all treatments and soil depths.

### 3.3. Relationships Among Soil Physicochemical Properties, Glomalin-Related Soil Proteins and Soil Aggregate Stability

**Figure 3:**
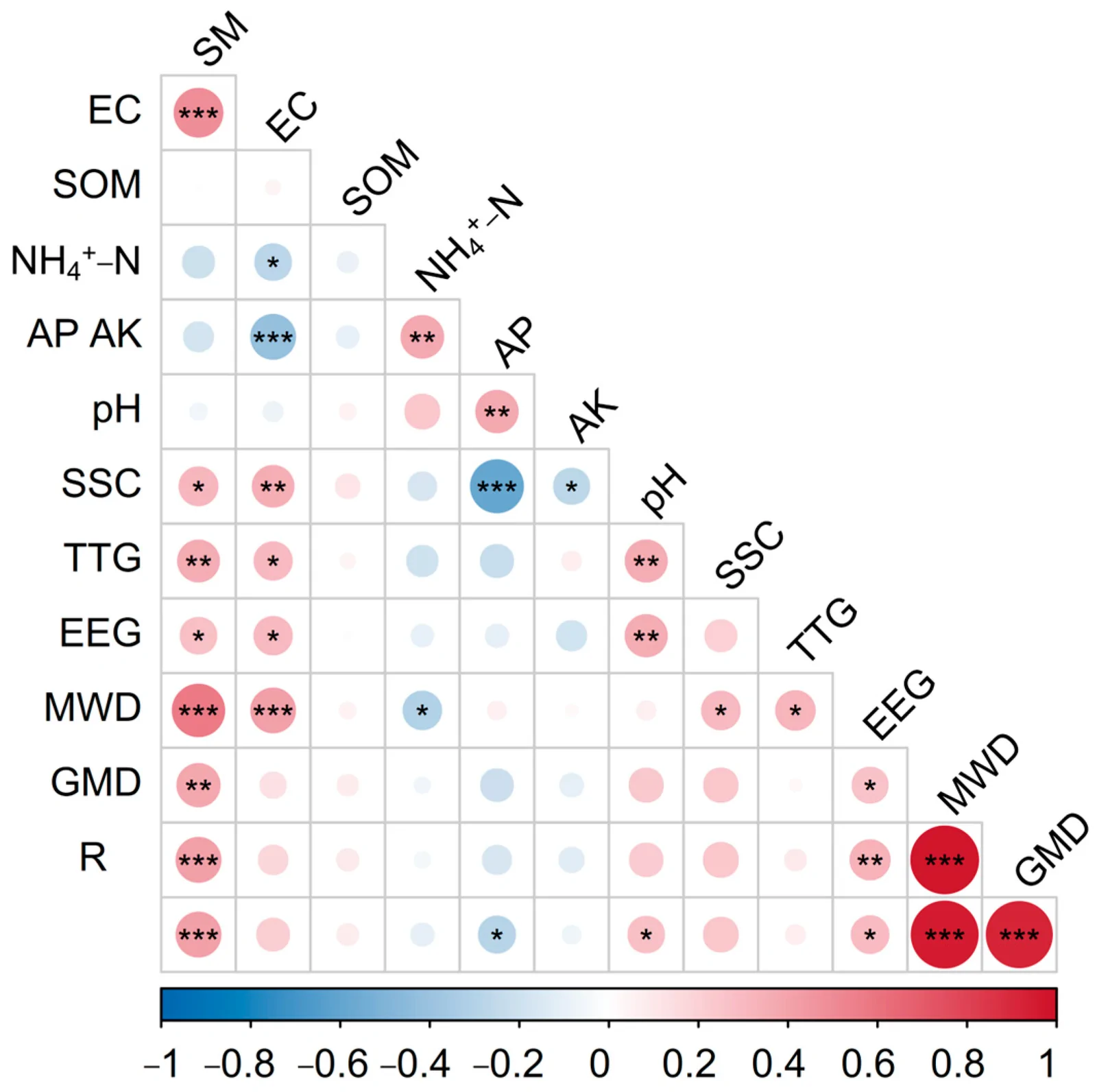
Pearson correlation matrix showing relationships among soil physicochemical properties, glomalin-related soil proteins, and soil aggregate stability. Note: Circle size and color represent the strength and direction of Pearson’s correlation coefficients, respectively. Red and blue circles indicate positive and negative correlations, respectively. A larger circle indicates a stronger correlation. Asterisks denote significant correlations (* *p* < 0.05, ** *p* < 0.01, *** *p* < 0.001). SM, soil moisture; EC, electrical conductivity; SOM, soil organic matter; NH_4_^+^-N, ammonium nitrogen; AP, available phosphorus; AK, available potassium; SSC, soil salt content; TTG, total glomalin-related soil protein; EEG, easily extractable glomalin-related soil protein; MWD, mean weight diameter; GMD, geometric mean diameter; R, percentage of water-stable aggregates.

Pearson correlation analysis revealed significant associations among soil physicochemical properties, glomalin-related soil proteins, and soil aggregate stability indices (Figure 3). SM was significantly and positively correlated with TTG, EEG, MWD, GMD, and R (*p* < 0.05), whereas EC showed significant negative correlations with EEG. TTG and EEG were significantly positively correlated with each other. Among the aggregate stability indices, MWD, GMD, and R were strongly and positively correlated with each other (*p* < 0.001). Regarding the relationships between GRSP and aggregate stability, EEG was significantly positively correlated with MWD, GMD, and R, while TTG showed significant positive correlations with GMD and R.

### 3.4. Structural Equation Model of Soil Aggregate Stability

**Figure 4:**
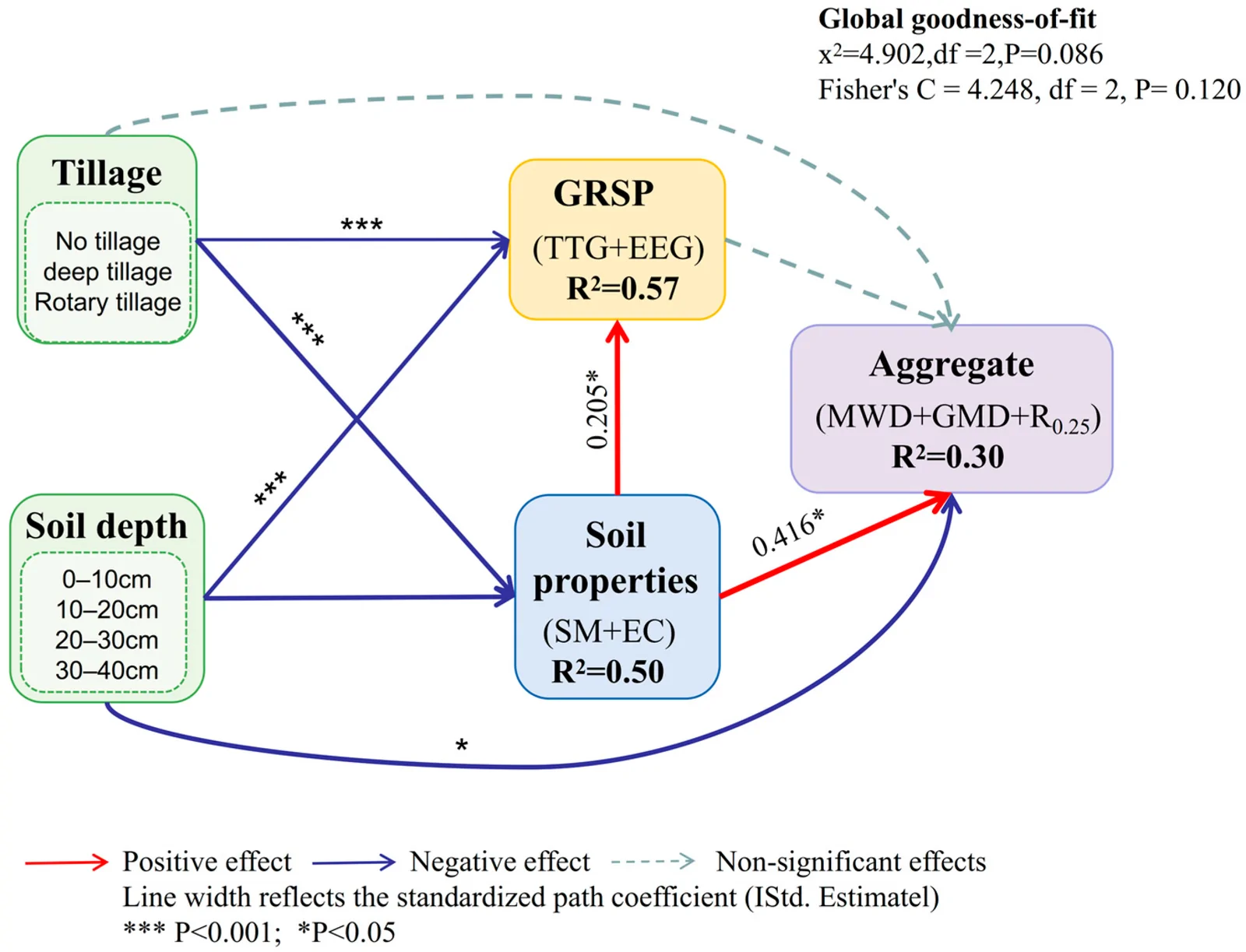
Structural equation model illustrating the direct and indirect pathways linking tillage practices, soil depth, soil properties, glomalin-related soil proteins, and soil aggregate stability. Note: solid arrows indicate significant pathways, whereas dashed arrows indicate non-significant pathways. Red and blue arrows represent positive and negative effects, respectively. Arrow width is proportional to the standardized path coefficient. Numbers adjacent to arrows indicate standardized path coefficients. Values inside the boxes represent the coefficient of determination (R^2^) for each endogenous variable. Model fit statistics are shown in the upper-right corner. Soil properties were represented by the first principal component (PC1) of soil moisture (SM) and electrical conductivity (EC). GRSP was represented by the first principal component (PC1) of total glomalin-related soil protein (TTG) and easily extractable glomalin-related soil protein (EEG). Aggregate stability was represented by the first principal component (PC1) of mean weight diameter (MWD), geometric mean diameter (GMD), and the percentage of water-stable aggregates (R). Significance levels are denoted as *** *p* < 0.001, * *p* < 0.05.

SEM was performed to evaluate the hypothesized relationships among tillage practices, soil depth, soil physicochemical properties, GRSP, and soil aggregate stability (Figure 4). Based on PCA results, SM and EC were selected as representative indicators of the soil physicochemical component because they showed strong contributions to the major environmental gradient. The model provided an acceptable fit to the observed data (χ^2^ = 4.902, *p* = 0.086; Fisher’s C = 4.248, *p* = 0.120), indicating that the proposed model adequately represented the covariance structure among variables. Tillage practices and soil depth significantly influenced soil physicochemical properties, explaining 50% of their variation (R^2^ = 0.50). Soil physicochemical properties showed a positive association with GRSP accumulation (standardized path coefficient = 0.205, *p* < 0.05), while tillage and soil depth also contributed directly to GRSP variation, resulting in a relatively high explanatory power (R^2^ = 0.57). Soil physicochemical properties further showed a positive direct effect on soil aggregate stability (standardized path coefficient = 0.416, *p* < 0.05). In contrast, the direct pathway from GRSP to aggregate stability was not statistically significant. Similarly, the direct pathway from tillage practices to aggregate stability was not statistically significant, whereas soil depth showed a significant direct pathway to aggregate stability.

## 4. Discussion

### 4.1. Influence of Tillage and Soil Depth on the Soil Physicochemical Environment

Tillage practices and soil depth jointly shaped soil physicochemical variation in the Mollisol agroecosystem, with dynamic soil environmental factors showing greater responses than relatively stable soil carbon pools. The relatively limited variation in SOM among treatments suggests that short- to medium-term changes in soil management may first be reflected in alterations of soil physical conditions and nutrient distribution rather than in rapid changes in bulk organic carbon storage. This pattern is consistent with previous studies showing that improvements in soil quality following tillage modification often occur initially through changes in soil structure and resource availability before detectable shifts in total carbon pools [38,39].

The PCA results further indicated that soil environmental variation was shaped by the combined effects of tillage practices and soil depth rather than by a single management factor. The partial separation and overlap among treatments suggest that tillage-induced changes occurred within a soil-depth-dependent environmental context. This finding highlights the importance of considering vertical soil heterogeneity when evaluating the effects of agricultural management, particularly in Mollisol regions where intensive cultivation has altered soil structure and resource distribution throughout the soil profile.

Soil moisture dynamics represent one of the most important environmental factors associated with tillage-induced changes in soil conditions. Conservation-oriented tillage systems generally improve water retention by minimizing soil disturbance, maintaining surface residues, and preserving soil pore continuity, whereas intensive mechanical disturbance may increase soil evaporation and reduce water storage capacity [40,41]. Deep tillage differs from surface disturbance because it may alleviate subsurface compaction and improve water movement within deeper soil layers [42]. Such changes in soil moisture conditions are particularly relevant in the black soil region of Northeast China, where seasonal water limitation is an important constraint on crop productivity. In addition, improved soil moisture conditions may provide a favorable environment for biological processes associated with GRSP accumulation and soil structural development.

Changes in electrical conductivity and pH further reflect the influence of tillage on soil environmental conditions. Variations in EC may be related to the redistribution of soluble ions and nutrients following soil disturbance, while pH responses may reflect differences in residue turnover and nutrient transformation processes under contrasting tillage systems. Similar changes in soil ionic conditions have been reported under different conservation tillage systems, where altered soil disturbance intensity affected nutrient redistribution and soil solution characteristics [42,43]. Although these changes were relatively moderate, shifts in soil chemical conditions can influence nutrient availability and create different environmental constraints for soil biological processes. Therefore, maintaining a balanced physicochemical environment is important for sustaining soil functions in intensively cultivated Mollisols.

The relatively stable SOM response observed across treatments is consistent with the slow turnover characteristics of soil organic carbon pools. Previous long-term studies have demonstrated that changes in soil carbon stocks often require extended periods because SOM represents an integrated outcome of carbon input, stabilization, and decomposition processes [44,45]. In contrast, changes in soil physical conditions and nutrient distribution can occur more rapidly following tillage modification. This difference suggests that improvements in soil structural quality under different tillage systems may precede measurable changes in bulk SOM [46].

Overall, these findings indicate that tillage practices were associated with changes in soil physicochemical conditions, while soil depth modified the magnitude and distribution of these responses throughout the soil profile. These environmental changes provide an important context for variations in GRSP accumulation and soil aggregate stability. The subsequent analyses further explored how soil physicochemical conditions were associated with biological indicators and soil structural characteristics under different tillage systems.

### 4.2. Responses of Glomalin-Related Soil Proteins and Soil Aggregate Stability to Tillage Practices

GRSP are widely recognized as important biological binding agents associated with soil aggregate formation and stabilization because they promote the adhesion of mineral particles and organic matter while enhancing resistance to physical disruption [47,48]. In the present study, tillage practices significantly influenced GRSP accumulation, particularly the EEG, whereas TTG exhibited relatively smaller variations among treatments. This difference likely reflects the contrasting ecological characteristics of the two GRSP fractions. EEG is considered a relatively dynamic fraction of GRSP that may respond rapidly to changes in soil environmental conditions and therefore responds rapidly to changes in soil environmental conditions, whereas TTG represents the cumulative accumulation of glomalin over longer periods and is consequently less sensitive to short-term management practices [49].

Compared with rotary tillage, deep tillage generally promoted greater GRSP accumulation, suggesting that improvements in the soil physical environment favored GRSP accumulation. Deep tillage reduces soil compaction and improves soil aeration and water movement, thereby creating more favorable conditions for root growth and GRSP-associated biological processes [50]. Previous studies have suggested that improved soil conditions and plant–soil interactions may contribute to GRSP accumulation under reduced soil disturbance; however, the specific biological processes involved require further investigation [51]. In contrast, repeated soil disturbance under rotary tillage may negatively affect GRSP accumulation by altering the soil environment required for biological stabilization processes. These findings agree with previous studies reporting that conservation-oriented tillage systems generally promote glomalin production by maintaining a more favorable soil environment [52].

The responses of GRSP were accompanied by corresponding changes in soil aggregate stability. Aggregate stability indices, including MWD, GMD, and R, were generally higher under deep tillage than under rotary tillage, indicating that improved soil conditions promoted the formation of more stable aggregates. This result is consistent with the concept that aggregate formation depends on the combined effects of biological binding agents and favorable soil physical conditions [53,54]. As a persistent glycoprotein, GRSP enhances aggregate stability by binding soil particles into larger structural units and protecting newly formed aggregates against mechanical breakdown [55]. Consequently, the similar response patterns observed for GRSP and aggregate stability suggest that biological stabilization contributed to the improvement of soil structure under appropriate tillage management.

However, the increase in aggregate stability did not always correspond proportionally with GRSP accumulation, indicating that glomalin is not the sole factor regulating aggregate formation. Soil aggregation is a complex process controlled by multiple interacting mechanisms, including soil organic matter, root exudates, microbial extracellular polymers, and clay–organic matter interactions [56,57]. Therefore, although GRSP represents an important biological component of aggregate stabilization, its influence should be interpreted together with changes in the soil physicochemical environment. This interpretation is consistent with our subsequent correlation analysis and structural equation modeling, which supported the close associations among soil properties, GRSP accumulation, and aggregate stability.

### 4.3. Associations of Soil Properties and GRSP with Soil Aggregate Stability

The correlation analysis provided further evidence that soil aggregate stability is jointly associated with soil physicochemical properties and biological processes rather than by individual environmental factors. Significant positive correlations were observed between SM, GRSP, and aggregate stability indices, whereas EC generally exhibited negative relationships with biological indicators. These results suggest that improvements in the soil physical environment create favorable conditions for GRSP accumulation and associated biological processes, thereby indirectly enhancing soil structural stability.

Among the measured physicochemical properties, soil moisture appeared to be one of the most influential environmental factors. Higher soil moisture was positively associated with TTG, EEG, and aggregate stability indices, indicating that adequate water availability promotes both microbial activity and aggregate formation. Soil moisture can influence nutrient transport and biological processes, all of which are important for root-associated biological processes and glomalin accumulation [58,59]. Previous studies have suggested that favorable soil water conditions may support biological binding processes, thereby strengthening aggregate stability [60,61]. Therefore, the positive association between SM and GRSP observed in the present study is consistent with the recognized role of water availability in regulating belowground biological processes.

In contrast, EC exhibited generally negative correlations with EEG and aggregate stability, suggesting that increasing concentrations of soluble ions may suppress biological activity under certain conditions. Although the EC values measured in the present study did not indicate severe salinity stress, elevatedion concentrations may still influence microbial communities through changes in osmotic potential and nutrient balance [62]. Similar negative relationships between soil salinity and AMF development have been reported in previous studies, where excessive salt accumulation reduced hyphal growth and limited glomalin production [63]. These observations indicate that maintaining an appropriate physicochemical environment is essential for sustaining biological processes associated with aggregate formation.

The strong positive correlations observed among TTG, EEG, and aggregate stability indices further support the important contribution of GRSP to soil structural development. GRSP has been widely recognized as a persistent biological adhesive that binds mineral particles and organic matter into stable macroaggregates [18,64]. In the present study, the consistent relationships between GRSP fractions and aggregate stability indicators suggest that GRSP accumulation was closely linked with improved soil structural characteristics. Nevertheless, aggregate formation is a complex process involving multiple interacting factors, including organic matter dynamics, mineral–organic interactions, and other biological binding components [65,66]. Therefore, the contribution of GRSP should be interpreted as part of a broader soil aggregation process rather than as an independent determinant of aggregate stability.

The strong relationships among MWD, GMD, and R confirmed that these indicators consistently reflected variation in soil structural quality. Similarly, the close associations between TTG and EEG supported the integration of these variables into composite indicators through PCA before SEM construction. This approach reduced model complexity while retaining the major ecological information represented by the original variables.

Although correlation analysis effectively revealed the associations among soil properties, GRSP, and aggregate stability, it could not distinguish direct from indirect pathways or quantify the relative contribution of each factor. Therefore, correlation analysis should be regarded as a preliminary assessment of variable relationships rather than definitive evidence of ecological mechanisms. To further clarify how tillage practices and soil depth regulate aggregate stability through changes in soil physicochemical properties and GRSP, structural equation modeling was subsequently applied to partition the direct and indirect effects among these variables.

### 4.4. Structural Equation Modeling Revealed the Relationships Associated with Soil Aggregate Stability

SEM provides an effective framework for disentangling the complex interactions among soil physicochemical properties, biological processes, and soil structural characteristics by simultaneously evaluating direct and indirect pathways [67]. Unlike conventional correlation analysis, SEM allows the integration of multiple variables into a conceptual model and evaluates whether the proposed relationships are consistent with the observed data. In the present study, the SEM showed an acceptable overall fit, indicating that the proposed conceptual model adequately described the relationships among tillage practices, soil depth, soil physicochemical properties, GRSP, and soil aggregate stability. The selection of SM and EC as representative indicators was intended to simplify the SEM structure and improve model interpretability rather than suggest that other soil properties were unimportant.

The SEM results indicated that soil physicochemical conditions were closely associated with variation in soil aggregate stability within the proposed model. Tillage practices and soil depth were associated with changes in soil physicochemical conditions, which were further related to variations in GRSP accumulation and aggregate stability. These findings suggest that changes in the soil environment may represent an important context associated with tillage-induced variation in soil structural characteristics. Similar studies have reported that soil moisture availability, nutrient conditions, and soil physical properties are closely linked with aggregate formation and stabilization processes [61,68]. Consequently, soil physicochemical properties should be regarded as the primary environmental driver linking agricultural management practices with soil structural quality.

The SEM did not support a statistically significant direct pathway from GRSP to aggregate stability, despite the significant correlations between GRSP and aggregate stability observed in the Pearson correlation analysis. This discrepancy suggests that their bivariate associations may partly reflect shared responses to soil environmental conditions rather than an independent contribution of GRSP to aggregate stability. Soil aggregation is a complex process involving multiple interacting physicochemical and biological components. Previous studies have shown that GRSP can contribute to soil aggregation through interactions with soil particles and organic matter, while other components, including organic matter, root-derived compounds, microbial products, and organo–mineral interactions, may also contribute to aggregate formation [65,69]. Similar conclusions have been reported in recent studies showing that GRSP often acts synergistically with other biological and physicochemical factors rather than functioning independently [70]. Accordingly, the non-significant GRSP pathway in the present SEM does not exclude a potential role of GRSP in soil aggregation but indicates that its independent contribution was not statistically supported within the structure of the current model.

Soil depth also showed significant relationships with soil aggregate stability within the SEM framework, highlighting the importance of vertical soil heterogeneity in agricultural ecosystems. Differences among soil layers may reflect variations in soil environmental conditions, resource availability, and structural characteristics along the soil profile. Previous studies have demonstrated that soil depth influences aggregate distribution and stability by altering the physical and chemical environment of different soil horizons [71]. Therefore, considering soil depth as an ecological factor rather than only a sampling category may improve our understanding of soil structural responses under different management practices.

The SEM also indicated that the direct pathway from tillage practices to aggregate stability was not statistically significant after accounting for soil physicochemical conditions, GRSP, and soil depth. In contrast, tillage practices were significantly associated with soil physicochemical properties, and soil physicochemical properties showed a significant positive pathway to aggregate stability. This pattern suggests that the observed differences in aggregate stability among tillage treatments may be associated with tillage-related variation in the soil physicochemical environment rather than with a direct effect of tillage alone. Deep tillage, for example, may modify soil physical conditions and resource distribution, potentially creating environmental conditions associated with changes in both GRSP accumulation and aggregate stability. This interpretation is consistent with previous studies showing that the ecological consequences of tillage depend not only on disturbance intensity but also on its effects on the broader soil environment.

Overall, the SEM supported a conceptual framework in which tillage practices and soil depth were associated with variations in soil physicochemical conditions, GRSP characteristics, and aggregate stability. These findings emphasize the importance of considering both abiotic and biological components when evaluating soil structural responses under different tillage systems. From a management perspective, practices that maintain favorable soil environmental conditions may contribute to improved soil structural quality in Mollisols. However, because AMF colonization, hyphal abundance, microbial community composition, and other biological processes were not directly measured, the biological mechanisms underlying GRSP responses and aggregate stabilization require further experimental validation.

### 4.5. Limitations and Future Perspectives

This study has several limitations that should be considered when interpreting the results. First, the experiment was conducted at a single field site under a continuous maize monoculture system; therefore, the observed responses of soil physicochemical properties, GRSP, and aggregate stability should not be directly extrapolated to crop rotation systems or other agricultural management contexts. Second, although GRSP was used as an indicator associated with biological contributions to soil aggregation, direct measurements of AMF colonization, fungal biomass, hyphal abundance, microbial community composition, and root traits were not included. Therefore, the biological mechanisms underlying GRSP variation and aggregate formation require further experimental validation. In addition, long-term multi-site experiments integrating crop rotation, microbial community analyses, AMF quantification, and root traits are needed to better understand soil structural development under diverse conservation tillage systems.

## 5. Conclusions

The present study provides new insights into the relationships among tillage practices, soil environmental conditions, glomalin-related soil proteins, and aggregate stability in the black soils of Northeast China. Deep tillage improved soil physicochemical conditions, particularly soil moisture and nutrient redistribution, and was associated with increased GRSP accumulation and enhanced soil aggregate stability compared with rotary tillage. Correlation analysis showed significant associations between GRSP and aggregate stability; however, the direct pathway from GRSP to aggregate stability was not statistically significant in the final structural equation model (SEM). Instead, soil physicochemical properties showed significant positive pathways to both GRSP and aggregate stability, suggesting that the observed covariation between GRSP and soil structural characteristics may partly reflect their shared responses to soil environmental conditions. Soil depth was also significantly associated with soil physicochemical properties, GRSP, and aggregate stability, highlighting the importance of vertical environmental heterogeneity in interpreting tillage-related responses.

These findings highlight that maintaining favorable soil physicochemical conditions is important for sustaining soil structural stability and soil quality in Mollisols. Appropriate tillage management may improve aggregate stability by optimizing soil environmental conditions and promoting GRSP accumulation. However, because AMF colonization, fungal biomass, hyphal abundance, microbial activity, and root-related processes were not directly measured in this study, the biological mechanisms underlying GRSP responses and aggregate formation require further experimental validation. Future studies integrating microbial community analyses, AMF quantification, root traits, and long-term field observations are needed to further elucidate the biological processes associated with soil aggregate formation under conservation tillage systems.

## Figures and Tables

**Table 1:** Soil physicochemical properties under different tillage practices and soil depths.

Variable	0–10 cm			10–20 cm			20–30 cm			30–40 cm		
	NT 0–10 cm	DT 0–10 cm	RT 0–10 cm	NT 10–20 cm	DT 10–20 cm	RT 10–20 cm	NT 20–30 cm	DT 20–30 cm	RT 20–30 cm	NT 30–40 cm	DT 30–40 cm	RT 30–40 cm
SM	16.11 ± 0.61 a	16.14 ± 1.41 a	12.38 ± 1.09 b	15.95 ± 1.52 a	15.63 ± 0.50 ab	13.69 ± 2.26 b	16.27 ± 0.90 a	15.85 ± 0.47 a	11.81 ± 2.43 b	17.05 ± 0.63 a	15.86 ± 1.11 a	16.24 ± 1.30 a
EC	104.88 ± 6.00 b	159.48 ± 41.90 a	63.08 ± 9.97 c	93.58 ± 9.70 a	67.72 ± 11.67 b	57.20 ± 20.64 b	141.84 ± 33.13 a	101.82 ± 7.80 a	105.96 ± 42.75 a	150.68 ± 9.55 a	111.36 ± 18.07 b	135.40 ± 29.62 ab
SOM	103.69 ± 0.36 a	103.80 ± 0.54 a	103.62 ± 0.13 a	104.17 ± 1.58 a	103.79 ± 0.47 a	103.63 ± 0.35 a	105.32 ± 4.46 a	103.37 ± 0.49 a	104.10 ± 0.29 a	104.33 ± 1.06 a	103.37 ± 0.30 b	104.13 ± 0.29 ab
NH_4_^+^-N	40.74 ± 21.84 a	33.79 ± 4.59 a	40.06 ± 5.37 a	43.49 ± 22.01 a	32.33 ± 5.01 a	50.55 ± 5.60 a	28.16 ± 2.41 a	29.92 ± 8.38 a	36.20 ± 8.47 a	28.95 ± 2.48 ab	20.21 ± 15.83 b	34.82 ± 8.00 a
AP	90.09 ± 0.38 a	89.92 ± 0.00 a	76.94 ± 28.57 a	88.24 ± 9.62 a	89.92 ± 0.00 a	77.82 ± 17.85 a	27.83 ± 11.21 a	58.99 ± 31.87 a	44.76 ± 26.89 a	20.97 ± 7.83 a	17.89 ± 10.54 a	13.55 ± 5.61 a
AK	243.76 ± 47.05 a	307.44 ± 139.96 a	197.84 ± 91.40 a	184.70 ± 72.14 b	239.18 ± 89.64 b	362.26 ± 87.09 a	207.98 ± 34.32 a	94.12 ± 54.94 b	213.56 ± 50.41 a	208.48 ± 58.42 a	198.74 ± 34.98 a	148.58 ± 51.94 a
pH	7.19 ± 0.30 a	6.13 ± 0.26 b	6.40 ± 0.36 b	7.00 ± 0.24 a	6.13 ± 0.51 b	6.21 ± 0.49 b	7.33 ± 0.34 a	6.68 ± 0.23 b	6.95 ± 0.47 ab	7.69 ± 0.29 a	7.34 ± 0.11 b	7.55 ± 0.11 ab
SSC	132.00 ± 14.30 a	111.00 ± 56.67 ab	79.50 ± 20.80 b	142.00 ± 28.80 a	143.00 ± 28.58 a	97.50 ± 19.76 b	147.00 ± 71.09 a	104.62 ± 15.35 a	128.00 ± 33.19 a	148.00 ± 67.44 a	123.50 ± 43.18 a	148.50 ± 26.79 a

**Note**: Values are means ± SD. Different lowercase letters indicate significant differences among treatments at *p* < 0.05. NT, no tillage; DT, deep tillage; RT, rotary tillage; SM, soil moisture; EC, electrical conductivity; SOM, soil organic matter; NH_4_^+^-N, ammonium nitrogen; AP, available phosphorus; AK, available potassium; SSC, soil salt content.

## Data Availability

The data are available from the corresponding author on reasonable request.

## Supplementary Materials

The following supporting information can be downloaded at: https://www.mdpi.com/article/10.3390/biology15161404/s1, Table S1: Effects of tillage practice, soil depth, and their interaction on soil properties, GRSP, and aggregate stability based on linear mixed-effects models.
