# Supplementary material for: Interactive Effects of Tillage and Soil Depth on Soil Aggregate Stability in Relation to Soil Properties and Glomalin-Related Soil Proteins
Source: Biology (Basel). 2026 Aug 16;15(16):1404. doi: 10.3390/biology15161404 (PMC13509733; doi:10.3390/biology15161404)
Supplement: Supplementary file 1 [file biology-15-01404-s001.zip › biology-4451876-supplementary.pdf]

Table S1. Effects of tillage practice, soil depth, and their interaction on soil properties, GRSP, and aggregate stability based on linear mixed-effects models

**Variable**	**Tillage F**	**P**	**Depth F**	**P**	**Tillage × Depth F**	**P**
SM	13.579	<0.001	7.158	<0.001	4.628	0.001
EC	7.869	0.007	17.893	<0.001	7.815	<0.001
SOM	1.111	0.361	0.468	0.706	0.619	0.714
NH_4^+-N	5.046	0.010	4.795	0.005	0.544	0.772
AP	1.646	0.234	69.745	<0.001	1.615	0.171
AK	0.494	0.613	5.663	0.002	5.025	<0.001
pH	15.820	<0.001	40.089	<0.001	1.907	0.106
TTG	6.750	0.011	6.743	0.001	1.305	0.280
EEG	49.735	<0.001	1.711	0.182	14.567	<0.001
SSC	2.796	0.071	1.667	0.186	1.158	0.344
MWD	3.685	0.057	3.304	0.031	0.384	0.884
GMD	6.472	0.012	2.581	0.069	0.585	0.740
R	4.183	0.042	3.631	0.022	0.029	1.000

Note: SM, soil moisture; EC, electrical conductivity; SOM, soil organic matter; NH_4^+-N, ammonium nitrogen; AP, available phosphorus; AK, available potassium; TTG, total glomalin; EEG, easily extractable glomalin; SSC, soil salt content; MWD, mean weight diameter; GMD, geometric mean diameter; R, percentage of water-stable aggregates. Tillage, soil depth, and their interaction were treated as fixed effects, whereas experimental plot was included as a random effect to account for the dependence among soil depths sampled within the same plot. F-tests were based on Satterthwaite's approximation for denominator degrees of freedom.
